# Remote Sensing of Wildland Fire-Induced Risk Assessment at the Community Level

**DOI:** 10.3390/s18051570

**Published:** 2018-05-15

**Authors:** M. Razu Ahmed, Khan Rubayet Rahaman, Quazi K. Hassan

**Affiliations:** Department of Geomatics Engineering, University of Calgary, Calgary, AB T2N 1N4, Canada; mohammad.ahmed2@ucalgary.ca (M.R.A.); krrahama@ucalgary.ca (K.R.R.)

**Keywords:** 2016 Horse River Fire, structural damages, very high spatial resolution, wildland-urban interface (WUI), WorldView-2

## Abstract

Wildland fires are some of the critical natural hazards that pose a significant threat to the communities located in the vicinity of forested/vegetated areas. In this paper, our overall objective was to study the structural damages due to the 2016 Horse River Fire (HRF) that happened in Fort McMurray (Alberta, Canada) by employing primarily very high spatial resolution optical satellite data, i.e., WorldView-2. Thus, our activities included the: (i) estimation of the structural damages; and (ii) delineation of the wildland-urban interface (WUI) and its associated buffers at certain intervals, and their utilization in assessing potential risks. Our proposed method of remote sensing-based estimates of the number of structural damages was compared with the ground-based information available from the Planning and Development Recovery Committee Task Force of Regional Municipality of Wood Buffalo (RMWB); and found a strong linear relationship (i.e., r^2^ value of 0.97 with a slope of 0.97). Upon delineating the WUI and its associated buffer zones at 10 m, 30 m, 50 m, 70 m and 100 m distances; we found existence of vegetation within the 30 m buffers from the WUI for all of the damaged structures. In addition, we noticed that the relevant authorities had removed vegetation in some areas between 30 m and 70 m buffers from the WUI, which was proven to be effective in order to protect the structures in the adjacent communities. Furthermore, we mapped the wildland fire-induced vulnerable areas upon considering the WUI and its associated buffers. Our analysis revealed that approximately 30% of the areas within the buffer zones of 10 m and 30 m were vulnerable due to the presence of vegetation; in which, approximately 7% were burned during the 2016 HRF event that led the structural damages. Consequently, we suggest to remove the existing vegetation within these critical zones and also monitor the region at a regular interval in order to reduce the wildland fire-induced risk.

## 1. Introduction

Wildland/forest fires are some of the critical natural hazards that pose a significant threat to the communities located in the vicinity of forested/vegetated areas around the world [1,2,3]. These fires, in fact, have an enormous impact on the urban/built environment adjacent to the wildland-urban interface (WUI). Those include: (i) interruption of usual activities of the residents [4]; (ii) destruction of buildings and other infrastructures [5]; (iii) evacuation of people [5,6]; (iv) degradation of air quality [6]; and (v) psychological traumatization of the people [7,8], among others. In the Canadian context, such fires impact 20 urban communities, 70,000 people, and forced the evacuation of about 8,500 people on an average per annum [9]. In fact, some of the largest wildland fires have occurred in the Western Canada (i.e., in the province of Alberta). For example, Lesser Slave Lake fire in 2011 (also known as the Flat Top complex fires) caused significant damages that include the destruction of 510 structures and evacuation of 15,000 residents [10] costing approximately $750 million in insurable damage [9]. More recently, on 1 May 2016, an enormous wildland fire in Fort McMurray (known as the Horse River Fire, HRF) destroyed 2579 dwelling units (1595 structures) and forced the evacuation of 88,000 people from the community [11]. Due to this fire event, the total estimated economic impact was about $8.9 billion, which represents the costliest insured natural disaster in the Canadian history [12]. In addition, Canada has observed an increasing trend in both the number of evacuations and evacuees as a result of the wildland fires occurred within the WUI over the period 1980–2014 [9,13]. Moreover, these numbers may potentially increase in the face of climate change [13]. Thus, it is critical for us to study the wildland fire-induced risk at the community level in order to formulate strategies to combat the adverse impacts. 

In comprehending the wildland fire-induce risks, one of the most important components is the characterization of the WUI. The detail information required for the appropriate characterization depends on the scale-level of the spatial dataset to be used for delineating WUIs, which eventually determine the effectiveness of the risk analysis [14]. In most of the instances, it is a common practice to delineate the WUIs from the spatial data at coarser details/small scales (e.g., at 1:50,000 or smaller), which typically represent country, regional, and continental levels [14]. In fact, such WUIs assessed from coarser spatial data suffer from several issues [14,15,16], such as: (i) they contain sharp boundaries; (ii) the edges of the classified land cover (including, urban, vegetation, and non-fuels) end abruptly; and (iii) fuels available inside the urban areas are not considered. Therefore, the adoption of these coarser spatial dataset for deriving WUIs is relatively less useful for community-level analysis [14,17,18,19], while they are the top-most impacted stakeholders in the event of an actual fire occurrence. Consequently, it is critical to generate the WUIs from the dataset at finer details/larger scales (e.g., at 1:10,000 or higher), which represent municipal, local, and community levels, and can potentially emphasize on fuel and wildfire hazard management issues at a micro level for the communities. In such cases, the most effective and accurate method is the employment of physical ground survey, since it could provide 1:1 scale data in greater details. However, it is not only very expensive, but also time-consuming, and a laborious process [20]. In this context, one of the viable alternates may be the use of high spatial-resolution (greater than 2 m) optical satellite data, which is a very much cost-effective, time-saving, and readily available technology.

In order to perceive wildland fire-induced risk at the community level, the comprehension of the damages within the WUI that took place during the historical wildfire events is essential. Such understanding would enable us to identify the connection of the inside urban fuels in the finer spatial dataset in WUI that helped to propagate the wildland fire towards the properties/structures. Additionally, this would help us to analyse the presence of wildland fuels, and structures in the vicinity of the WUI. For example, Syphard et al. [21] used finer spatial data (i.e., housing/structure footprints) in their fire risk modelling studies. In the scope of damage assessment, there are several methods, such as, physical ground survey-, airborne remote sensing-, and spaceborne remote sensing-based methods. Although the physical ground survey method can provide the most detailed and accurate assessment, it suffers similar limitations as described in the previous section for mapping WUI. The second method is the use the airborne remote sensing that includes the aerial photography, and unmanned aerial vehicle (UAV)-based imaging. Some of the such noteworthy case studies included: (i) the Regional Municipality of Wood Buffalo (RMWB) [22] in Alberta, Canada employed aerial-based photographs known as Pictometry imagery taken from five different views (i.e., east, west, north, south, and top) for the Fire Assessment Tool (FAT) to assess the damages occurred due to the HRF in the Fort McMurray urban service area in 2016; (ii) Galarreta et al. [23] analysed UAV imageries of Grounau (Germany), Enschede (The Netherlands), and Bologna (Italy) for urban structural damage assessment. They used oblique imagery taken from multi-angles and 3D point-clouds to develop an automatic damage assessment tool/procedure upon implementing object-based image analysis and semantic reasoning. The study delineated damages related to facades and roofs with aggregated damage score and certainty-measurements; and (iii) Pham et al. [24] combined aerial photographs with Light Detection and Ranging (LiDAR) data to map structural damages in 2010 at Haiti. In reality, the use of airborne technology has the advantages of proving very high spatial-resolution (usually higher than 0.3 m) imagery that facilitates assessing detailed damages of each individual structure. However, the acquisition and processing of such aerial imagery are quite costly, and time-consuming procedure.

In order to overcome the limitations of airborne remote sensing, spaceborne remote sensing (i.e., satellite imagery) can be used in minimizing the cost associated with the image acquisition, and reducing the time for information extraction. Some of the example case-studies include: (i) United Nations High Commissioner for Refugees [25] observed a massive fire destruction in Myanmar using WorldView satellite platforms with a spatial resolution of 0.3–0.5 m. Their study determined that 275 towns and villages were destroyed or otherwise damaged by fire in Buthidaung, Maungdaw, and Rathedaung Townships in the Maungdaw and Sittwe districts of Rakhine State; (ii) Ehrlich et al. [26] assessed the damages in Wenchuan, China by employing both very high resolution (VHR) optical satellite imageries (i.e., EROS-B, WorldView-1, and SPOT-5) and SAR satellite imageries (i.e., CosmoSkymed and TerraSAR-X). They demonstrated the effectiveness of using such imagery for quantifying building stock and assessing damages; and (iii) Platt [27] employed VHR WorldView-2 and LiDAR imageries for mapping wildfire hazard of the home ignition zone (HIZ, a small area of the WUI) in the west of Boulder (Colorado, USA). In the study, damaged structures (included in the burned area) were mapped with an overall accuracy of 90% total agreement with the ground truth data.

In the scope of this paper, our overall goal was to use WorldView-2 imagery with a spatial-resolution of 0.5–2 m to develop a wildland fire-induced risk modelling framework upon considering the 2016 HRF over Fort McMurray (Alberta, Canada). There were two specific objectives. Firstly, we estimated the number of structural damages due to the wildland fire of interest by using high spatial resolution satellite images, and compared them against ground-based information available from the Planning and Development Recovery Committee Task Force of RMWB. Secondly, we delineated WUI using the satellite images, and generated buffer zones at 10 m, 30 m, 50 m, 70 m and 100 m from the WUI. Subsequently, we used them in quantifying their impacts in damaging the structures and formulating risk zonation induced by the wildland fire at the community-scale.

## 2. Study Area and Data Requirements

### 2.1. General Description of the Study Area

**Figure 1:**
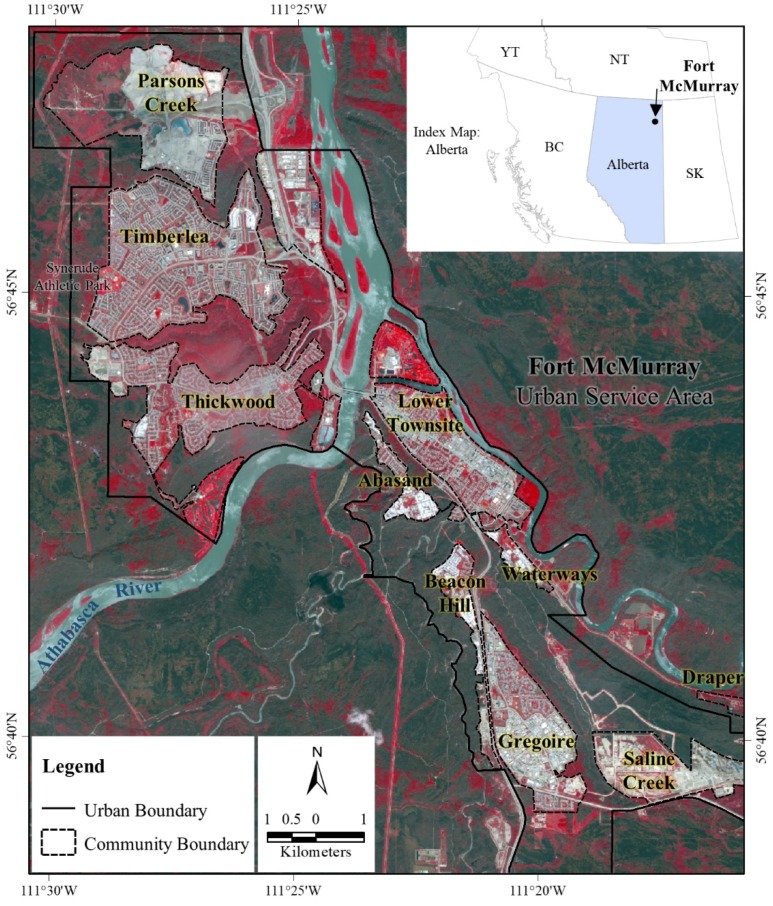
The spatial extent of the study area highlighting the ‘Fort McMurray Urban Service Area’ and community boundaries using solid and dotted polygons respectively using a WorldView-2 satellite image acquired on 6 June 2016; which is located in the northeastern part of the Province of Alberta. Note that the urban area is surrounded by both burned (as seen in dark greenish to gray colors) and healthy (bright red to reddish colors) vegetation.

We considered Fort McMurray as our study area (Figure 1), which is a self-contained ‘urban service area’ located on the banks of the Athabasca River in the northeastern part of Alberta, Canada. This service area is situated at a distance of approximately 450 km northeast from the nearest metropolitan city Edmonton. The population of Fort McMurray is approximately 66,573 [28], which has grown significantly (i.e., from 1186 to 61,374) over the period 1961–2011 [29]. Such population growth has been observed as a result of developing oil sands resources in the Greater Athabasca Region. This region, in fact, is the third largest confirmed oil deposits in the world; and covers an area of approximately 140,000 km^2^ [30]. Being the center of the oil sands industry, Fort McMurray dominates the local to national economy that contributes to the 30% of the total regional labor force [31].

The climate of Fort McMurray is considered severe during winters, and mild to warm and dry summers for only three months. The daily average temperature in January and July for the Fort McMurray during 1981–2010 were recorded −17.4 and +17.1 °C, respectively [32]. The region is characterized by a borderline subarctic climate, which is very close to the humid continental climate. The annual average precipitation of the area is 418.6 mm during the 1981–2010 period [32]. The physiography of the area is considered as ‘Northern Alberta Lowlands’ [33], which is having an average elevation of 369 m above mean sea level [34]. The area falls under the ‘Central Mixedwood’ natural subregions of Alberta [33], which is surrounded by thick boreal forest with a mix of bog and oil sands with dense coniferous covering [35]. Due to the presence of hefty boreal forest coupled with drier summer, the ‘Central Mixedwood’ natural subregion has experienced significant amount of wildland fires, i.e., 9323 number of lightning-caused and 9101 number of human-caused fires; which constituted as approximately 34.37% of the total wildland fire incidents in Alberta during the 1961–2014 period [36].

### 2.2. Data Requirements

In this study, we primarily used three key datasets: (i) spaceborne WorldView-2 satellite imagery received from DigitalGlobe Foundation; (ii) historical imageries available from Google Earth Pro system; and (iii) statistical ground data of the damaged structures from RMWB after the fire event in 2016. In case of the WorldView-2 imagery, it was acquired on 6 June 2016 (i.e., post wildland fire event) in both panchromatic (Pan, spatial resolution 0.5 m) and multispectral (MS, spatial resolution 2.0 m) modes. The Pan band of WorldView-2 covers spectral wavelengths of 0.45–0.80 µm. In contrast, the WorldView-2 MS provides eight multispectral bands that includes, coastal (0.40–0.45 µm), blue (0.45–0.51 µm), green (0.51–0.58 µm), yellow (0.585–0.625 µm), red (0.63–0.69 µm), red edge (0.705–0.745 µm), near infrared-1 (0.77–0.895 µm), and near infrared-2 (0.86–0.90 µm) [37]. However, we employed four visible and near infrared (VNIR) MS bands (i.e., blue, green, red, and NIR1) for mapping and assessing structural-damages in this study. Note that we used only the four VNIR bands instead of eight bands available with WorldView-2 imagery, since most of the VHR satellite imagery contain at least these four bands (see the study that used QuickBird imagery having four VNIR-bands for mapping structural-damage due to the 2011 wildfire in Slave Lake, Alberta, Canada [38]). Additionally, we utilized a combination of Pan and MS bands coupled with historical imageries of Google Earth Pro for the characterization of WUI and the risk modelling for the pre-during-post events of the wildland-fire in Fort McMurray. In addition, we obtained the ground-based data in relation to the damaged structures; which were gathered and analysed by the RMWB Planning and Development Recovery Committee Task Force, and reported in McIntyre [11]. Furthermore, we acquired additional information from RMWB (Allison Kennedy-Drake, Performance & Risk Analyst of Recovery Task Force, personal communication) for perceiving the basis of counting the number of structural loss in different structural arrangements on the ground. Such additional information included: (i) multiple dwelling units or townhouses with a single and continuous rooftop was counted as one structure-loss or multiple structure loss; (ii) a damaged unattached-garage was counted as one structure-loss or not; (iii) a business facility with multiple structures was considered as multiple count or a single count of loss, etc. We, then, used this information in validating the remote sensing-derived estimates of the damaged structures. Finally, we gathered more information regarding the community street maps, structural plans and Municipal Development Plan of RMWB [39,40], and the community protection guidelines of Canadian FireSmart [41,42]; used them both in delineating community boundaries and modelling risks associated with wildland fire. 

## 3. Methods

**Figure 2:**
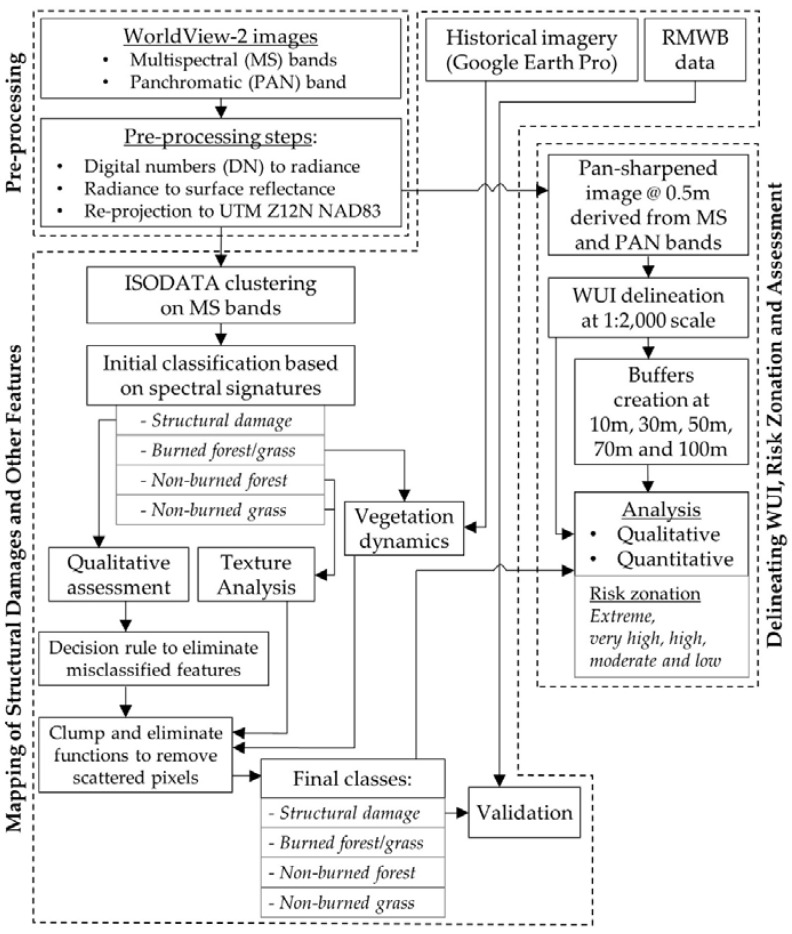
Schematic diagram of the proposed methods for mapping structural damages and zoning wildland-induced risk areas at the communities of Fort McMurray.

Figure 2 shows the schematic diagram of our proposed methods. It consisted of three major components: (i) pre-processing of WorldView-2 images; (ii) mapping of structural damages and other features; and (iii) delineating WUI, risk zonation and assessment using pan-sharpened WoridView-2 images. These components are briefly described in the following sub-sections.

### 3.1. Pre-Processing of WorldView-2 Images

Upon acquiring the WorldView-2 satellite images, we performed the following pre-processing steps on both Pan and MS bands of interest (i.e., VNIR). Those included: (i) converting the digital numbers (DN) of the pixels in the images to radiance values (Equation (1)) [43,44]; (ii) transforming the radiance values to surface reflectance values (Equation (2)) [43,45]; (iii) re-projecting the images into Universal Traverse Mercator (UTM) Zone 12 N with North American Datum 1983 (NAD 83); and (iv) clipping the images to the extent of the study area. Further, we used this pre-processed dataset in mapping of the spatial dynamics due to the HRF in Fort McMurray area as described in the following sub-section.

(1) $$L_{\lambda }=G*DN+B$$

(2) $$\rho_{\lambda }=\frac{\pi *\left[L_{\lambda }-L_{\lambda }\left(haze\right)\right]*d^{2}}{ESUN_{\lambda \;}*\;\cos\theta_{s}}$$

where *L_λ_* and *ρ_λ_* are the radiance and surface reflectance, *DN* is the digital number, *G* and *B* are the gain and bias values, *L_λ_*(*haze*) is the minimum radiance value in the histogram, *d* is Earth-Sun distance in astronomical units, *ESUN_λ_* is mean solar exoatmospheric irradiances, and *θ_s_* is solar zenith angle. All these parameters are band-specific, and the values of *G*, *B*, *d*, *ESUN_λ_*, and *θ_s_* are available from the metadata files of the images.

### 3.2. Mapping of Structural Damages and Other Features

In delineating the features of interest, i.e., “structural damage”, “burned forest/grass”, “non-burned forest” and “non-burned grass” over the study area, we applied ISODATA (i.e., iterative self-organizing data analysis technique) clustering technique to the WorldView-2 MS image. Our preference of using this clustering technique was due to its ability of statistically attributing each and every pixel of an image to generate specific number of classes based on the spectral similarities [45]. In this process, we generated 50 classes with a convergence threshold of 0.995 by assigning an infinite number of iterations. Subsequently, we evaluated the patterns of cluster-specific spectral signatures, and grouped them according to our features of interest. 

In case of mapping “structural damage”, we consulted additional datasets, such as historical imageries acquired between August 2015 and May 2016 available from Google Earth Pro and Fire Map of RMWB [22]. At this stage, we appraised a qualitative assessment and noticed few large buildings (mostly with flat rooftops) with no fire-induced damages were misclassified as damaged structures. To address this issue, we defined such areas and applied a decision rule of declaring them as non-damaged structures. Additionally, we observed some scattered misclassified pixels, which were then removed by applying ‘clump’ and ‘eliminate’ functions. Finally, we counted the number of damaged structures, and validated them with the ground-based information synthesized by the RMWB Planning and Development Recovery Committee Task Force [6]. In this case, we determined the degree of agreements between the satellite- and ground-based estimates of structural damages by percentage error at the community-level.

For the “burned forest/grass”, “non-burned forest” and “non-burned grass”, we conferred with the available historical imageries from Google Earth Pro as mentioned above in order to comprehend the vegetation dynamics during the pre-event of HRF. We observed that some burned forest/grass and non-burned forest in the southern portion of the Thickwood neighborhood were misclassified as “non-burned grass”. This was, in fact, due to the presence of haze-effect on that part of the image. Thus, we identified the extent of the haze areas and implemented a decision rule to assign the “non-burned grass” to “burned forest/grass” and “non-burned forest” as appropriate. Additionally, for “non-burned grass”, we recognized that some areas were misclassified as “non-burned forest”. To sort out this issue, we performed a texture analysis, and applied a decision rule to recode the misclassified forest as “non-burned forest” having rough texture, and as “non-burned grass” having smooth texture. We eventually used these three feature-classes in the wildland fire-induced risk zonation and discussed further in the following sub-section.

### 3.3. Delineating WUI, Risk Zonation and Assessment

In order to delineate risk zones, and assess the presence/absence of vegetation (i.e., fuels) within these zones, we employed a combination of WorldView-2 Pan and MS data. In this case, we accomplished such integration using a pan-sharpening technique known as subtractive resolution merge [46,47]. In fact, such technique would enhance the visualization, and also allow observing finer details on the pan-sharpened image [48,49]. Note that we did not use this pan-sharpened image for delineating spatial features as detailed in Section 3.2, because the pan-sharpening would affect the original spectral quality provided by the multispectral bands [50,51]. 

In delineating the WUI, we visualized the pan-sharpened image at 1:2000 scale and drew the interface upon considering the property-lines adjacent to the wildland. Note that we drew property-lines as the interface, because the property-lines in the communities were typically bounded by wood fence that were connected to the structures. This finer details or large-scale, in fact, aided visualizing spatial features in such a detail, which was not possible using the Community Street Maps of Fort McMurray available at 1:10,000 scale [38]. It would be worthwhile to mention that we delineated structure- or building-based WUI, which would be a common practice for mapping finer spatial data for WUI from high resolution imagery [52,53]. Next, we generated three outside-buffers of 10 m, 30 m, and 100 m from the WUI, which were the Interface Priority Zones (IPZ) described in the guidebook for community Protection of FireSmart Canada [42,54] for managing vegetation around WUI. Upon generating the buffers, we assessed the presence of different fuels (i.e., forest and grass in particular) and their on-ground standing arrangements (i.e., clear-cut, thinning, and pruning) within each of the buffer zone for three different time-frames, i.e., before, during, and after the HRF. At this stage, we consulted with the historical imageries acquired between August 2015 and May 2016 available from Google Earth Pro for the assessment of standing-vegetation that potentially posed risk for the communities before and during the wildland fire event. During the aftermath of the fire event, we used the spatial features (that included “structural damage”, “burned forest/grass”, “non-burned forest” and “non-burned grass”) as described in Section 3.2. While assessing the risks for the three buffer-zones, we perceived that additional buffer zones would be very useful for assessing potential risks at the community level. Hence, we generated additional two buffer zones from the WUI, i.e., 50 m and 70 m for providing recommendations in order to reduce the wildland fire-induced risks for the communities in the study area. Note that we used two approaches for assessing the risk zones, such as: (i) qualitative assessment that involved visual examination; and (ii) quantitative analysis for assessing potential risk areas. For the quantitative analysis, we quantified the presence of on-stand forest/trees (i.e., non-burned forest class in our classification) within each of the five buffer zones, and categorized into five classes. The classes were extreme risk, very high risk, high risk, medium risk, and low risk based where on-stand forest/trees were existed within WUI to 10 m, 10 to 30 m, 30 to 50 m, 50 to 70 m and 70 to 100 m buffer area respectively. We considered those areas as very low risk, which were greater than 100 m away from WUI or where there were no presence of standing forest/trees between the WUI and 10 to 100 m buffer zones.

## 4. Results and Discussion

### 4.1. Structural Damage Assessment

**Figure 3:**
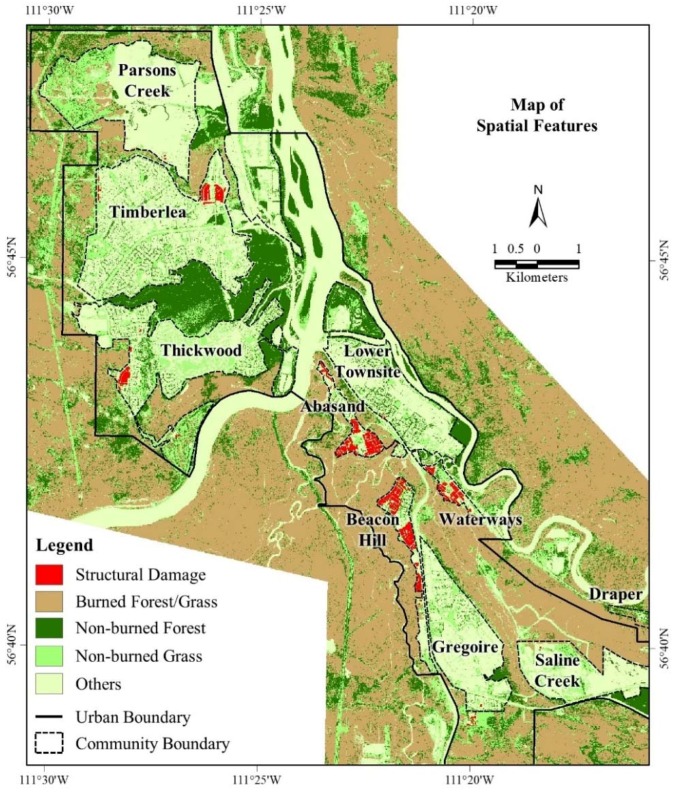
The spatial extent of the structural damage derived from WorldView-2 MS satellite image acquired on 6 June 2016 including other spatial features of interest.

**Figure 4:**
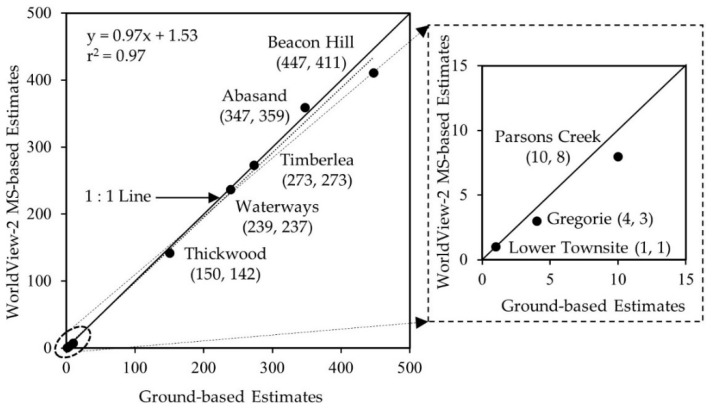
Relationships between the structural damage estimates using satellite- and ground-based counts.

Figure 3 shows the WorldView-2 MS derived structural damages, including other features, i.e., burned forest/grass, non-burned forest and non-burned grass over the communities of the study area. We found that the highest number of structural damages occurred in the communities of Beacon Hill (411 structures), and Abasand (359 structures). In contrast, we estimated that the lowest structural damage took place in the community of Lower Townsite (1 structure), which was followed by Gregoire (three structures) and Parsons Creek (eight structures). We compared the number of structural damages derived from our proposed method with the ground-based estimates at the community level (Figure 4). Note that we considered only eight communities (i.e., Abasand, Beacon Hill, Gregoire, Lower Townsite, Parsons Creek, Thickwood, Timberlea, and Waterways), which were completely covered by the satellite image. However, we were unable to estimate such damages over other communities within the Wood Buffalo Region, because those areas were either covered partially (e.g., Draper, and Saline Creek) or not at all (e.g., Anzac, Saprae Creek, and Fort McMurray International Airport) by the satellite image. In general, we observed a very good relationship (i.e., r^2^ = 0.97 with a slope and intercept of 0.97 and 1.53, respectively) between satellite-based remote sensing and ground-based estimates. In most cases, our estimates were either at par or slightly below except for the community of Abasand. The probable rationales of such under estimates were as follows:

- The satellite image provided only the top view, i.e., rooftops of the structures; which was unable to provide any further information related to damages occurred in the side-walls of the structures. As a result, we failed to identify such damaged structures.
- In the areas with the presence of both large and small houses together (e.g., Beacon Hill North, Beacon Hill South, and Waterways), it would be possible that we counted few small houses as detached garages. Also, note that we didn’t count the damaged detached garages as separate structures, rather than included as part of the main structures.
- In case of the community of Beacon Hill, our count difference was the highest (i.e., ground-based estimates of 447 vs. remote sensing-based estimates of 411). Other studies also reported similar count differences, e.g., Hassan et al. [38]. In addition to the above-mentioned causes, there might have another reason. In the southern part of the community (i.e., Centennial RV Park at around the Latitude 56°40′47″ N and Longitude 111°21′13″ W), we observed an informal arrangement of several damaged structures, which were probably a campground having temporary shelters of tiny and linear houses, trucks, or caravans. In this case, we did not include those small damages in our estimates, rather counted only the two permanent structures located in the area.

Note that we also found two damaged structures in Dickinsfield (near the intersection of McConachie Crescent and Clenell Crescent) of the Thickwood community, which were burned after the HRF event. Thus, we didn’t include them in our count of fire-induced structural damages. In addition, in the community of Abasand, we had over estimations, i.e., ground-based estimates of 347 vs. remote sensing-based estimates of 359. It might be attributed due to the following reasons:

- We might have counted some detached garage as separate structure in the dense built-up locations, where the boundaries of the houses were not clearly distinguishable from the satellite image;
- It would be quite possible that some of the town-houses were continuous. However, we were unable to identify such connectivity; thus, interpreted and counted as separate structures; and
- It was quite challenging to comprehend the utilization of the structures from the satellite data, hence a business with multiple structures would possibly be counted as multiple structural damages. Note that one business operation with several structures were considered as one structure in the ground-based estimates (Allison Kennedy-Drake, Performance & Risk Analyst of Recovery Task Force; personal communication).

### 4.2. Delineation of WUI and Buffers, and Assessment of Potential Risks

#### 4.2.1. Qualitative Assessment

**Figure 5:**
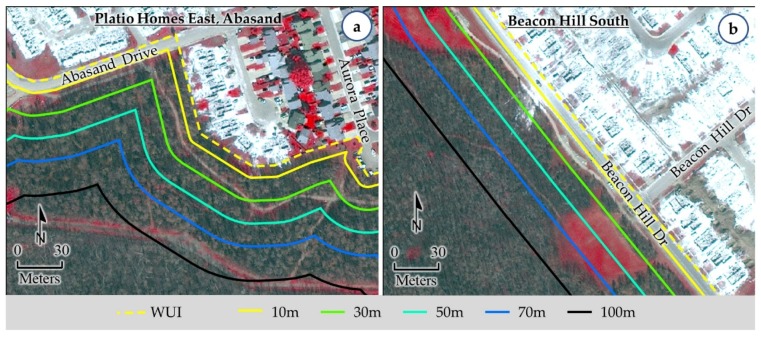
Example of areas of wildland fire-induced structural damages where there was presence of vegetation (fuel for fire propagation) within 10 m (panel **a**) and 30 m (panel **b**) buffers from the WUI.

As per the methods discussed in Section 3.2, we contrived the WUI and its associated buffers at 10 m, 30 m, 50 m, 70 m, and 100 m. Subsequently, we investigated two specific issues, i.e., (i) relation with the WUI and its associated buffers and fire-induced structural damages; (ii) delineation of the spatial dynamics of the wildland fire-induced vulnerable area. In case of the areas with observed structural damages, we found that there was presence of vegetation (fuel for the fire; see Figure 3 for burned forest/grass, and non-burned forest and grass) within the 10 m buffer from the WUI in most of the instances (see Figure 5a for an example case). Furthermore, we noticed the existence of vegetation within the next 20 m from the 10 m buffer (i.e., 30 m buffer from the WUI) for the remaining areas of structural damages (see Figure 5b for an example case). Thus, we might infer that the zone of 30 m buffer should have very little to no existence of vegetation in order to avoid the propagation of wildland fire into the communities.

**Figure 6:**
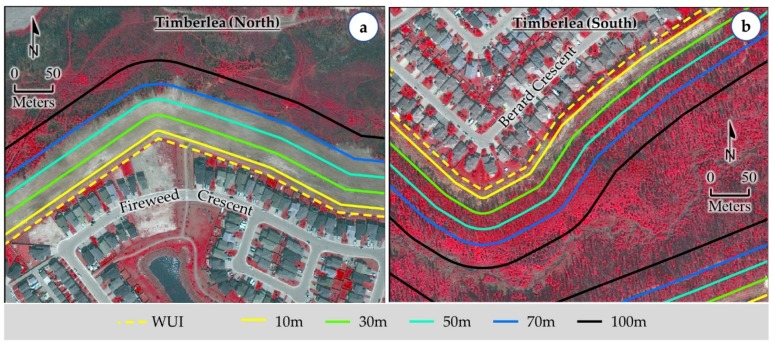
Example of areas with guided vegetation removal in order to protect nearby communities such as Timberlea panel (**a**) in particular. Panel (**b**) shows an example of wildland fire-induced vulnerable area.

It would be interesting to note that we observed the removal of vegetation between 30 m and 70 m buffers from the WUI (see Figure 6a for an example). In fact, we assumed that such operation was carried out by relevant authorities in order to protect the structures in the nearby communities; which was evident upon consulting very high spatial resolution satellite imageries available from Google Earth Pro acquired on 3, 4, 5, 7, and 12 May 2016. From this observation, we might emphasis that a vegetation zone of up to 70 m from the WUI would enhance the safety measures in order to reduce the wildland fire-induced risk. Thus, our delineation of additional buffer zones of 50 m and 70 m from the WUI would be effective and helpful in formulating better wildland fire-induce risk mitigation strategies. In addition, we also looked into the wildland fire-induced vulnerable areas upon considering the WUI and its associated buffers. We found that there were still some communities that had the existence of vegetation within the buffer zones (see an example in Figure 6b). In such circumstances, we would recommend that the vegetation should be completely removed within 10 m buffer zone of WUI as also recommended by FireSmart guidelines [41,42,54]. Additionally, though the guidelines prescribed for thinning and pruning of vegetation for 10–30 m buffer zone, we would like to suggest complete removal of dense vegetation from the zone, which was an example case observed during the HRF event in the northern side of Timberlea community (see Figure 6a). Also, in order to reduce the wildland fire-induced risks, it would be critical to have a regular monitoring system in place to assess the vegetation conditions and remove them if deemed necessary. 

#### 4.2.2. Quantitative Assessment

**Figure 7:**
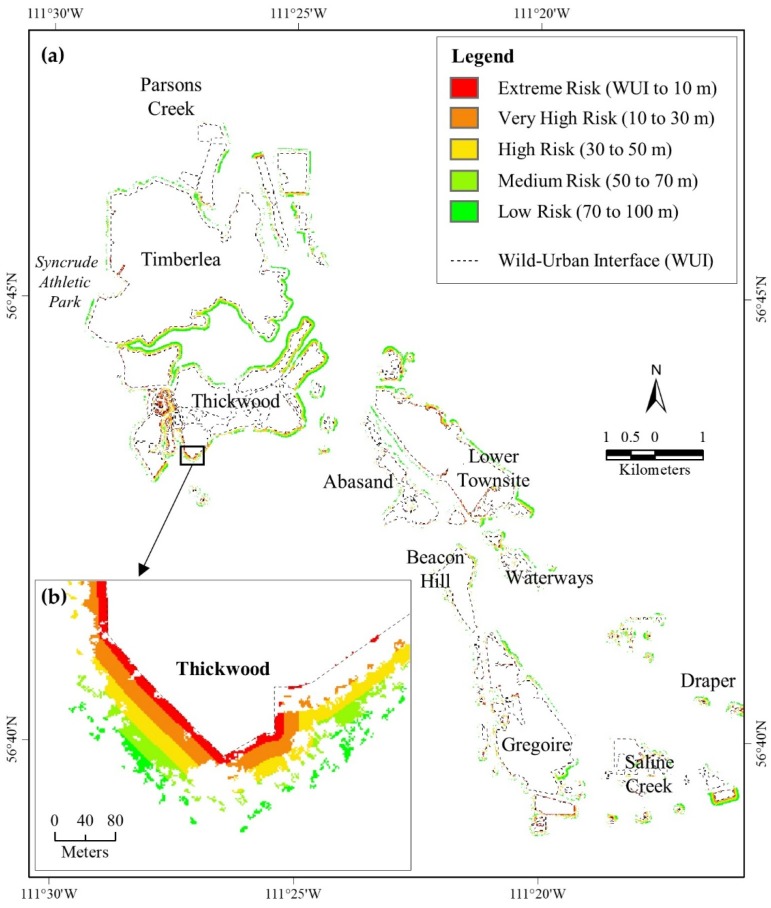
Wildland fire-induced risk areas for the communities of Fort McMurray identified by quantitative analysis [panel (**a**)]; Panel (**b**) shows an example in large scale for different categories of risk existed in the south of Thickwood community.

According to FireSmart guidelines [41,42,54], there should be complete clear-cut within WUI to 10 m buffer zone. However, our quantitative analysis revealed that 10.35% areas were under ‘extreme risk’ (Table 1) for the associated communities for having on-stand forest/trees on 6 June 2016. We also found that 11.34% and 18.18% areas were under ‘very high risk’ and ‘high risk’ respectively for the associated communities (see column A in Table 1). Additionally, presence of standing fuels during the HRF event (i.e., combining our classified “burned forest/grass” and “non-burned forest” classes) were found 12.62% within WUI to 10 m zone (see column D in Table 1), which also indicated that area as ‘extreme risk’ category. The ‘very high risk’ and ‘high risk’ categories were also having 17.28% and 28.52% of standing fuels respectively during the HRF event (Table 1). Spatial locations of the risk categories in the communities are shown in Figure 7.

Our analysis revealed that damaged structures were associated with the presence of greater available fuels during the HRF event within the adjacent buffer zones (i.e., 37.94%; see column B_1_ in Table 1) in compared to non-damaged structures (i.e., 15.42%; see column B_2_ in Table 1). It was observed that approximately 30% (i.e., a total of ‘extreme risk’ and ‘very high risk’ categories described in column D in Table 1) of the areas within the buffer zones of 10m and 30m were vulnerable due to the presence of vegetation during the 2016 HRF event. In which, approximately 7% (i.e., a total of ‘extreme risk’ and’ very high risk’ categories of column B_1_ in Table 1) in the two buffer zones (i.e., 10 m and 30 m) were burned that led the structural damages. It would be worthwhile to note that initial ignitions to the structures that eventually caused further structural damages were most likely from embers or radiant heat of the burning vegetation in the buffer zones [55].

### 4.3. Other Considerations

Apart from the delineation of structural damages in communities and assessment of vulnerabilities due to the HRF, we also summarized a few interesting evidences while evaluating the developed models and risk zonation (i.e., buffers in this particular case). In case of Syncrude Athletic Park, we discerned that the area was situated at the western part of Timberlea community (Figure 1). There were some fuel connections to expand the wildland fire into the Athletic Park’s compound and so did towards the nearest community. However, due to having some open space with very little vegetation, wildland fire did not spread out to other communities; where the Athletic Park itself worked as a major obstacle to fire propagation. Through this experience, we assumed that similar major social service infrastructures (e.g., elementary schools); recreation activities (athletic parks, golf courses); shopping malls (e.g., Walmart, Canadian Tire), and major highways (wider than 40 m including the right of ways) could be planned at the outskirts of the urban service area to create fuel-break in future development. We also would suggest that considerations should be given priority to erect the parking lots for shopping malls, playgrounds, churches, golf courses, schools, etc. that would fetch the vegetated areas as a major barrier for wildland fire spread. Also, we would like to suggest that the land use plans should incorporate the wildland fire-vulnerability information if not included in the current practice.

In addition, other dynamic factors including weather, topography, and vegetative stages are also critical in defining the wildland fire-induced risks. In this context, it would be worthwhile to note that the Earth Observation Laboratory at the University of Calgary had developed primarily remote sensing-based forest fire danger forecasting system capable of producing daily to 8-day level danger/risk conditions [56,57,58,59]. In these cases, the set of variables included: (i) Moderate Resolution Imaging Spectroradiometer (MODIS)-based 8-day composites of surface temperature (Ts), normalized multiband drought index (NMDI: an indicator of canopy moisture conditions) and temperature vegetation wetness index (TVWI: an indirect measure of soil water content) [56,58]; (ii) MODIS-based 8-day composites of Ts, NMDI and normalized difference vegetation index (NDVI: a measure vegetation greenness) [57,58]; (iii) MODIS-derived 8-day composites of Ts, NDVI and NMDI, and daily precipitable water (PW) [59]; and (iv) Shuttle Radar Topography Mission (SRTM)-derived digital elevation model to calculate topographical elements including elevation, slope, and aspect [60]. Despite the importance of these dynamic factors, we opted not to include them in the scope of this study; which would be incorporated in further studies depending on the available resources. 

## 5. Concluding Remarks

In the scope of this paper, we demonstrated the effectiveness of using Worldview-2 in developing a wildland fire-induced risk modelling framework and applied for the comprehension of the 2016 HRF occurrence in Fort McMurray, Alberta. We found that the estimates of the fire-induced structural damages using satellite- and ground-based information exhibited strong linear relations, i.e., r^2^-value of 0.97 with a slope of 0.97. We also observed that vegetation was available within the 10 to 30 m buffers from the WUI, which thought to be the reason of initial ignitions of the fire and eventually responsible for further propagation causing structural damages. Furthermore, our consultation with the very high spatial resolution satellite images available from the Google Earth Pro acquired between 3 and 12 May 2016 (that coincided with the HRF duration) revealed that the relevant authorities had removed vegetation in some critical areas between 30 and 70 m buffers in order to protect the structures in the nearby communities. Also, upon mapping the wildland fire-induced vulnerable areas using the WUI and its associated buffers, we spotted that the existence of dense vegetation within the 10 to 30 m buffer zones around some communities, which would be removed to minimize wildland fire-induced risks. Despite our findings, we strongly recommend that our proposed methods should be carefully evaluated and modified accordingly (if deemed necessary) prior to adopting in other ecosystems.

## Figures and Tables

**Table 1:** Quantitative analysis and risk categories based on the presence of tree-standing in each buffer zone in Fort McMurray.

Risk Category	Buffer Zone (m)	Area under Potential Risk (%)	Area of Burned Forest/Grass (i.e., Fuels Adjacent to the Structures) (%)			Tree Standing during HRF (%)
		(A)	(B)		(C)	(D)
			Damaged	Non-Damaged	Total	(A + C)
			(B_1_)	(B_2_)	(B_1_ + B_2_)	
Extreme Risk	WUI to 10	10.35	1.43	0.84	2.28	12.62
Very High Risk	10 to 30	11.34	5.82	0.12	5.94	17.28
High Risk	30 to 50	18.18	8.10	2.24	10.34	28.52
Medium Risk	50 to 70	22.40	9.12	5.30	14.42	36.83
Low Risk	70 to 100	26.07	13.47	6.92	20.39	46.46
	Total		37.94	15.42		
